# P-1823. Hepatitis B Status and COVID-19 Severity of Adult Admitted Patients in a Tertiary Hospital in the Philippines

**DOI:** 10.1093/ofid/ofaf695.1992

**Published:** 2026-01-11

**Authors:** Angeli Eleanor Facun, Bernard Demot, Marie Ellaine Velasquez

**Affiliations:** Baguio General Hospital and Medical Center, Baguio CIty, Benguet, Philippines; Baguio General Hospital and Medical Center, Baguio CIty, Benguet, Philippines; Baguio General Hospital and Medical Center, Baguio CIty, Benguet, Philippines

## Abstract

**Background:**

Several prevalence studies have shown that the Philippines is endemic for Chronic Hepatitis B with estimated 16.7% of the Filipino adult population and that COVID-19 can co-infect with Hepatitis B with a prevalence between 0-1.3%. COVID-19 has a multi-organ involvement that can be associated with disease severity and mortality risk. Currently, there are scarce and contradicting results in the available data regarding patients with Hepatitis B and COVID-19.

**Figure:**
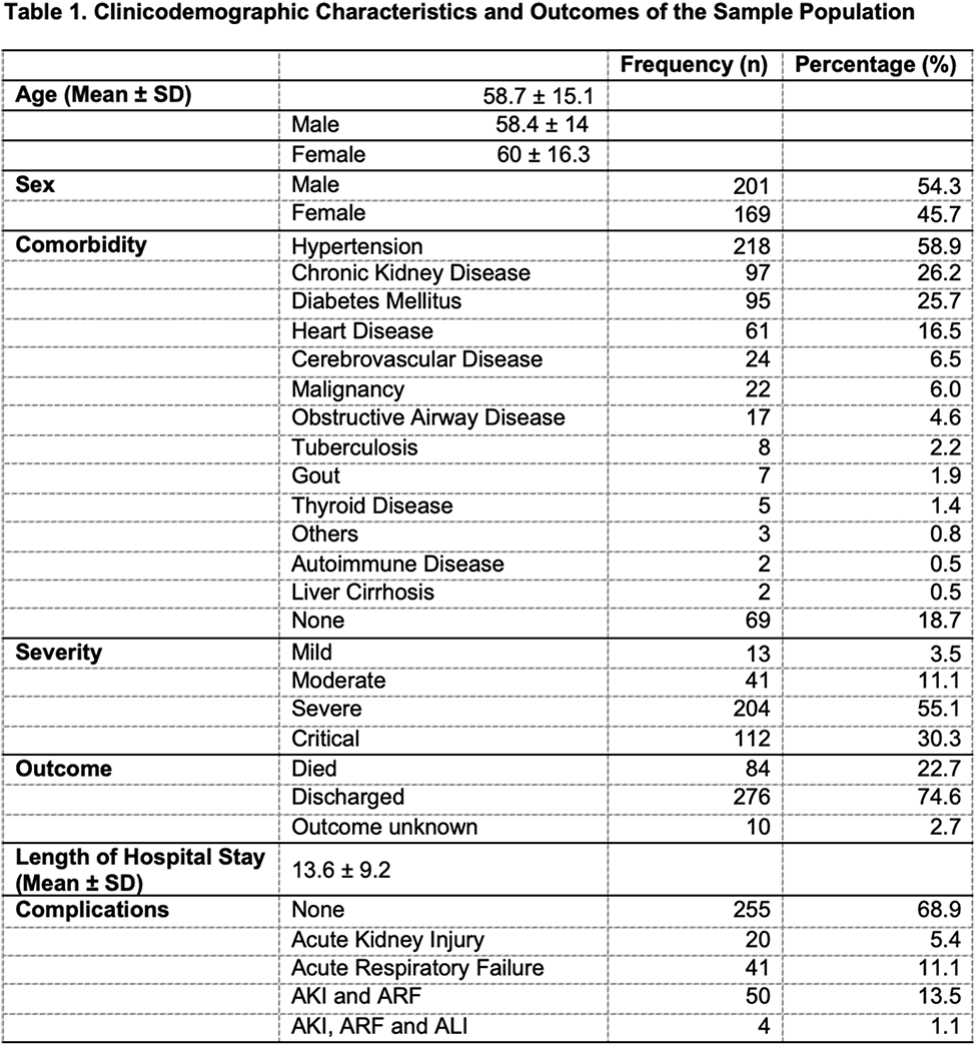

**Figure:**
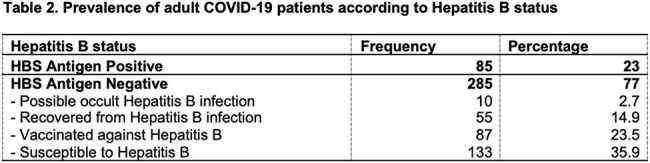

**Methods:**

This is a cross-sectional descriptive and analytic study involving adult COVID-19 patients with a sample size of 370 and a confidence level of 99%. Descriptive statistics in terms of frequencies and percentages were utilized for the categorical variables. Chi-square was used to determine associations between Hepatitis B status and COVID-19 severity as well as to compare patients in terms of clinical outcomes and complications whereas, two-way ANOVA was used to compare means with an alpha level of significance of < 0.05.

**Figure:**
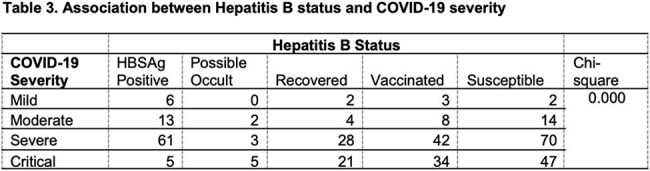

**Figure:**
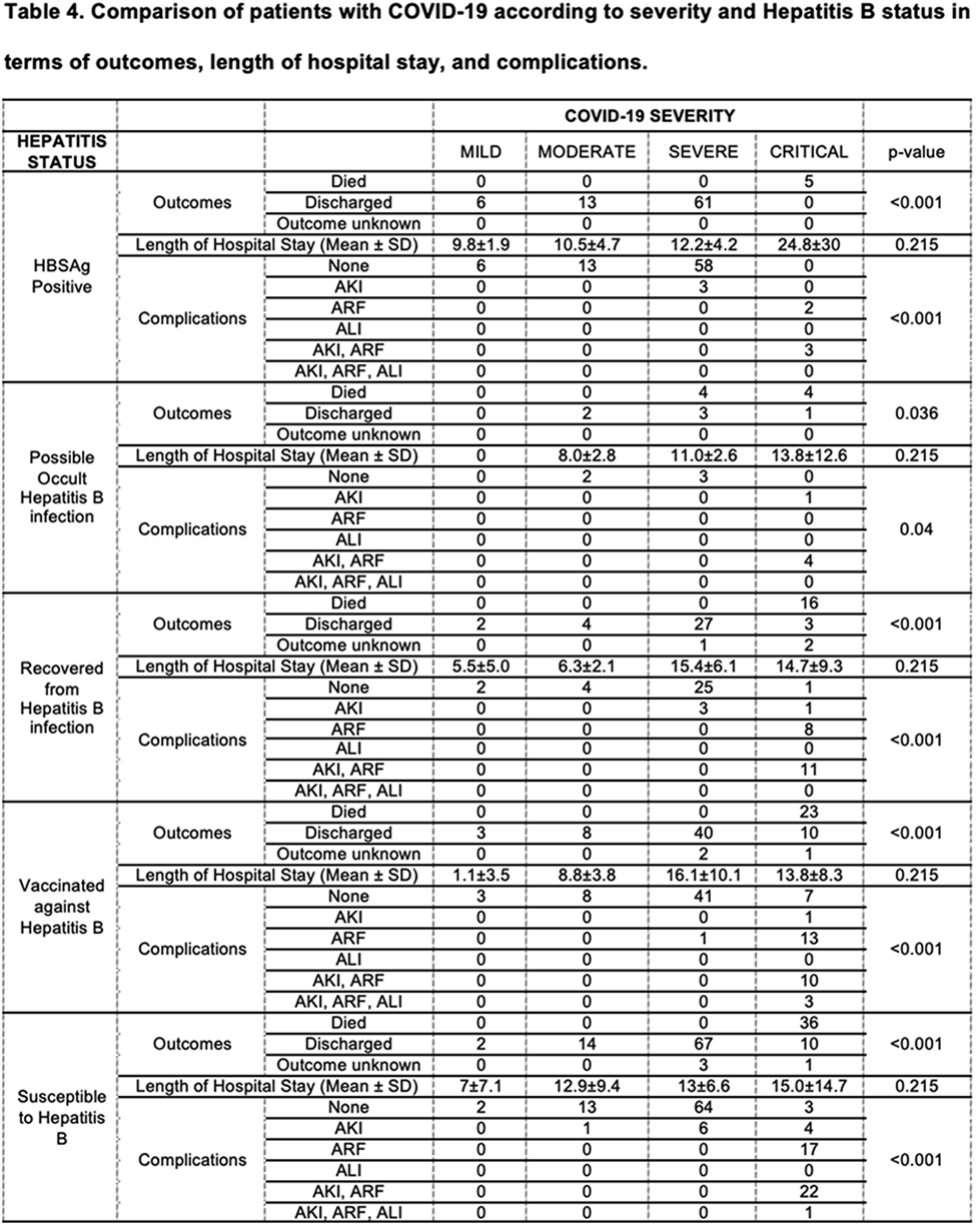

**Results:**

The clinicodemographic profile of patients reveals majority having severe COVID-19 disease (55.1%), critical (30.3%), moderate (11.1%) and mild COVID-19 disease (3.5%). Twenty-three percent (23%) are HBSAg positive. In contrast, 77% are HBSAg negative, of which 35.9% are susceptible to Chronic Hepatitis B, 23.5% are vaccinated against hepatitis B, 14.9% are recovered from hepatitis B infection, and 2.7% have possible occult hepatitis B infection. There is a significant association between Hepatitis B classification and COVID-19 severity. There is a significant relationship between COVID-19 severity and hepatitis B status in terms of clinical outcomes and complications, but none in terms of the length of hospital stay.

**Conclusion:**

Hepatitis B is identified as a disease with high burden in the Philippines. This study reflects the need to concentrate and direct more effort on testing and vaccination on this subset of vulnerable populations, especially during the pandemic.

**Disclosures:**

All Authors: No reported disclosures

